# Nephrotic syndrome caused by IgA vasculitis flare up following COVID-19 vaccination

**DOI:** 10.1093/qjmed/hcad040

**Published:** 2023-03-15

**Authors:** T Horino, Y Osakabe, M Ishihara, K Nakajima, Y Terada

**Affiliations:** Department of Endocrinology, Metabolism, and Nephrology, Kochi Medical School, Kochi University, Kohasu, Oko-cho, Nankoku, Kochi 783-8505, Japan; Department of Endocrinology, Metabolism, and Nephrology, Kochi Medical School, Kochi University, Kohasu, Oko-cho, Nankoku, Kochi 783-8505, Japan; Department of Paediatrics, Kochi Medical School, Kochi University, Kohasu, Oko-cho, Nankoku, Kochi 783-8505, Japan; Department of Dermatology, Kochi Medical School, Kochi University, Kohasu, Oko-cho, Nankoku, Kochi 783-8505, Japan; Department of Endocrinology, Metabolism, and Nephrology, Kochi Medical School, Kochi University, Kohasu, Oko-cho, Nankoku, Kochi 783-8505, Japan

Learning points for clinicians The coronavirus disease 2019 (COVID-19) mRNA vaccine can cause flare ups of glomerulonephritis, especially in patients with IgA vasculitis. The reporting of relevant cases can help us examine and understand the mechanisms of disease onset following COVID-19 vaccination, and thus, implement measures to prevent the recurrence or exacerbation of such diseases.

A 64-year-old female patient presented to our nephrology clinic with haematuria, proteinuria and palpable purpura on both lower legs. She had been treated for immunoglobulin A (IgA) vasculitis for 10 years from the age of 7 and had been in long-term remission without treatment thereafter. One month prior to her visit, she had experienced fever and gross haematuria the day after she received the fourth dose of the BNT162b2 mRNA coronavirus disease 2019 (COVID-19) vaccine. Several days later, the fever and gross haematuria subsided, but purpura appeared on both lower legs. The patient visited a local dermatologist who referred her to our clinic. Physical examination showed pitting oedema and palpable purpura on both lower legs. Serum creatinine was 0.84 mg/dl on admission, which increased to 1.05 mg/dl by hospital Day 7 and was 1.65 mg/dl on hospital Day 12. Urinalysis revealed 20 red blood cells per high power field and granulocytic casts in the urine sediment. Urine protein excretion was 6.7 g/gCr. A renal biopsy specimen collected on hospital Day 7 showed mild mesangial cell and mesangial matrix proliferation, endocapillary hypercellularity lesions (Figure 1A), cellular crescents (Figure 1A, Supplementary Figure S1A) and glomerular necrosis with basement membrane damage and leakage of plasma components (Figure 1A, Supplementary Figure S1B). Immunofluorescence microscopy findings revealed dominant IgA mesangial staining (Figure 1B) with C3. Based on the above findings, the patient was diagnosed with nephrotic syndrome and acute kidney injury caused by IgA vasculitis flare up following COVID-19 vaccination. Treatment with intravenous methylprednisolone (1 g/day) for 3 days was initially administrated, followed by oral prednisolone (40 mg/day). The symptoms and laboratory abnormalities resolved without recurrence.

**Figure 1.**
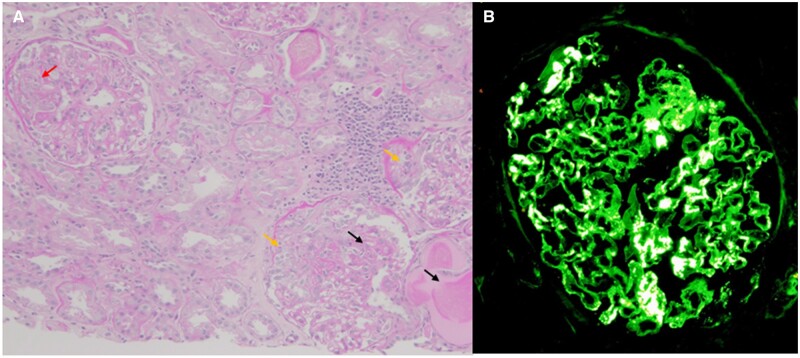
Histological findings of the renal biopsy specimen. (**A**) Light microscopy findings showed mild mesangial cell and mesangial matrix proliferation, endocapillary hypercellularity lesions (red arrow), cellular crescents (yellow arrows) and glomerular necrosis with basement membrane damage and leakage of plasma components (black arrows) (Periodic acid-Schiff stain, original magnification 200×). (**B**) Immunofluorescence microscopy findings showed dominant IgA mesangial staining (original magnification 400×).

It is widely known that vaccines induce immune responses that can lead to various autoimmune diseases.^1^ Although COVID-19 vaccination has been recommended since the start of the COVID-19 pandemic, there are still many uncertainties regarding the associated risks. As vaccination rates increase, various new-onset or recurrent glomerular and immune-mediated diseases, such as pericarditis, IgA vasculitis, myasthenia gravis, multiple sclerosis and arthritis,^2^ following COVID-19 mRNA vaccination have been reported.^3^ Anti-severe acute respiratory syndrome (SARS) coronavirus antibodies have been reported to react with 28 tissue antigens from diverse tissue groups, including barrier proteins, gastrointestinal, thyroid and neuronal tissue^4^; and thus, COVID-19 vaccination may have the potential to induce autoimmune reactions. An association between increased anti-SARS-CoV-2 spike IgA after COVID-19 vaccination and reactivation of pre-existing IgA vasculitis has also been reported.^5^ Renal pathological findings in our case included prominent mesangial proliferative changes, intracapillary proliferation, glomerular necrosis/rupture and crescent formation. This supports the speculation in previous reports that focal rupture of the glomerular basement membrane due to intraductal proliferation is a major precipitating factor in early or acute exacerbation of IgA nephropathy and IgA vasculitis.^6^ The detection and investigation of patients with new-onset IgA nephropathy or IgA vasculitis after COVID-19 vaccination provide an opportunity to understand underlying mechanisms of these diseases. Activation of multiple autoimmune or autoinflammatory systems by vaccination can cause serious diseases that otherwise would not have developed or recurred, which can have serious impacts on the patient's life and organ prognoses. Our accumulated scientific knowledge about COVID-19 vaccines is achieved through the reporting of relevant cases. Together, these cases enable us to examine the mechanisms leading to disease onset following vaccination, and thus, implement measures to prevent the occurrence of serious side effects.

## Supplementary Material

hcad040_Supplementary_Data

## Data Availability

Data are available upon reasonable request by any qualified researchers, who engage in rigorous, independent scientific research and will be provided following review and approval of a research proposal and Statistical Analysis Plan (SAP) and execution of a Data Sharing Agreement (DSA). All data relevant to this study are included in the article.
