# Supplementary figures and images for: Nephrotic syndrome caused by IgA vasculitis flare up following COVID-19 vaccination
Source: QJM. 2023 Mar 15;116(7):556–8. doi: 10.1093/qjmed/hcad040 (PMC10382190; doi:10.1093/qjmed/hcad040)

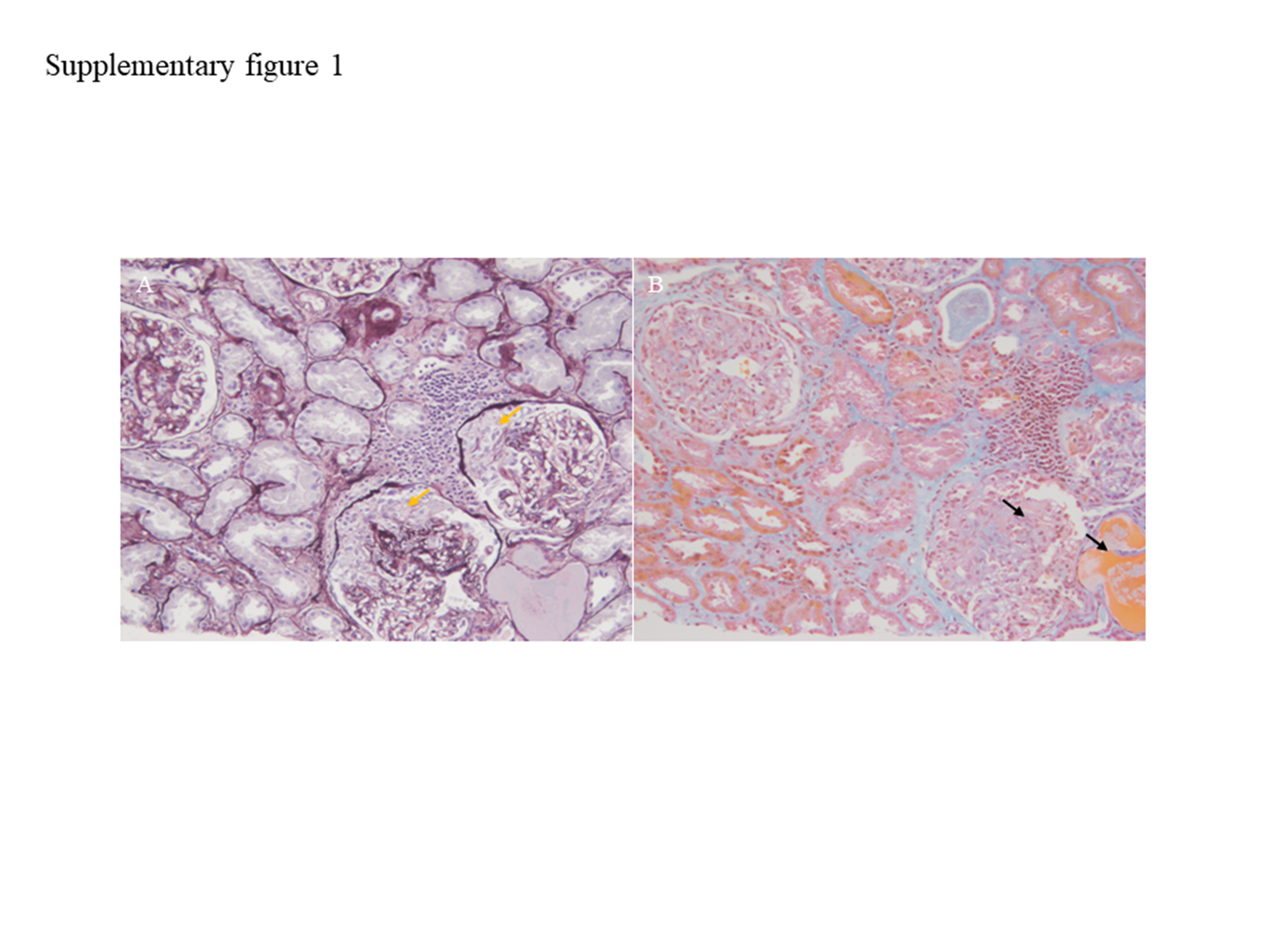

Supplement: hcad040_Supplementary_Data [file hcad040_supplementary_data.zip › hcad040_Supplementary_Data/hcad040_Supplementary_Fig-1.TIF]
